# Spontaneous, Pro-Arrhythmic Calcium Signals Disrupt Electrical Pacing in Mouse Pulmonary Vein Sleeve Cells

**DOI:** 10.1371/journal.pone.0088649

**Published:** 2014-02-20

**Authors:** Katja Rietdorf, Martin D. Bootman, Michael J. Sanderson

**Affiliations:** 1 Department of Microbiology and Physiological Systems, University of Massachusetts, Medical School, Worcester, Massachusetts, United States of America; 2 Department of Life, Health and Chemical Science, The Open University, Milton Keynes, United Kingdom; 3 Signalling Programme, The Babraham Institute, Cambridge, United Kingdom; SUNY College of Nanoscale Science and Engineering, United States of America

## Abstract

The pulmonary vein, which returns oxygenated blood to the left atrium, is ensheathed by a population of unique, myocyte-like cells called pulmonary vein sleeve cells (PVCs). These cells autonomously generate action potentials that propagate into the left atrial chamber and cause arrhythmias resulting in atrial fibrillation; the most common, often sustained, form of cardiac arrhythmia. In mice, PVCs extend along the pulmonary vein into the lungs, and are accessible in a lung slice preparation. We exploited this model to study how aberrant Ca^2+^ signaling alters the ability of PVC networks to follow electrical pacing. Cellular responses were investigated using real-time 2-photon imaging of lung slices loaded with a Ca^2+^-sensitive fluorescent indicator (Ca^2+^ measurements) and phase contrast microscopy (contraction measurements). PVCs displayed global Ca^2+^ signals and coordinated contraction in response to electrical field stimulation (EFS). The effects of EFS relied on both Ca^2+^ influx and Ca^2+^ release, and could be inhibited by nifedipine, ryanodine or caffeine. Moreover, PVCs had a high propensity to show spontaneous Ca^2+^ signals that arose via stochastic activation of ryanodine receptors (RyRs). The ability of electrical pacing to entrain Ca^2+^ signals and contractile responses was dramatically influenced by inherent spontaneous Ca^2+^ activity. In PVCs with relatively low spontaneous Ca^2+^ activity (<1 Hz), entrainment with electrical pacing was good. However, in PVCs with higher frequencies of spontaneous Ca^2+^ activity (>1.5 Hz), electrical pacing was less effective; PVCs became unpaced, only partially-paced or displayed alternans. Because spontaneous Ca^2+^ activity varied between cells, neighboring PVCs often had different responses to electrical pacing. Our data indicate that the ability of PVCs to respond to electrical stimulation depends on their intrinsic Ca^2+^ cycling properties. Heterogeneous spontaneous Ca^2+^ activity arising from stochastic RyR opening can disengage them from sinus rhythm and lead to autonomous, pro-arrhythmic activity.

## Introduction

Throughout a typical human lifetime, a coordinated ‘cardiac cycle’ of atrial and ventricular contraction is repeated over a billion times [1]. The cardiac cycle is initiated by the sino-atrial node (SA node) located within the right atrial wall. The SA node generates action potentials (APs) that sweep over the atrial and ventricular chambers to cause them to contract and pump blood [2]. This contraction is mediated by Ca^2+^ increases within cardiac myocytes via the well-known process of ‘excitation-contraction coupling’ (EC-coupling). Essentially, membrane depolarisation opens voltage-operated Ca^2+^ channels (VOCCs) to allow Ca^2+^ influx into the ‘dyadic’ cleft between the sarcolemma and the sarcoplasmic reticulum (SR; the myocyte Ca^2+^ store). This Ca^2+^ signal is amplified by Ca^2+^-induced Ca^2+^ release via ryanodine receptors (RyRs) on the SR, thus causing a global Ca^2+^ signal that triggers actin-myosin filament interaction and myocyte contraction [3]. After each AP, myocytes become refractory to further electrical stimulation for tens to hundreds of milliseconds, and during this time Ca^2+^ is returned to diastolic levels by Ca^2+^-ATPases on the sarcolemma and SR, and Na^+^/Ca^2+^ exchangers on the sarcolemma [4]. In humans, the normal sinus rhythm is ∼60 beats per minute, but during atrial fibrillation (AF) the atrial chambers can display activity in excess of 300 beats per minute due to aberrant electrical signals [5]. Because the ventricular chambers are largely responsible for pumping blood, AF is not immediately life threatening, but a loss of atrial function can lead to fainting and chest pain. Moreover, blood clotting resulting from stagnant or turbulent blood in the atrial chambers greatly increases the risk of stroke [6].

It is believed that increased ‘automaticity’ (spontaneous depolarisation of myocytes), acute ‘triggered activity’ (spontaneous electrical events following recovery from an action potential) or ‘re-entry circuits’ (return of an electrical impulse to cardiac cells following a refractory period) contribute to AF. The underlying causes of all these pro-arrhythmic conditions are not fully understood, but substantial evidence has implicated spurious Ca^2+^ signals as a likely cause [6]–[8]. Spontaneous Ca^2+^ signals occurring during the recovery from a previous AP, or during the quiescent diastolic phase, can depolarise the sarcolemma and potentially trigger an ectopic AP or alter the refractoriness of myocytes relative to their neighbours [9].

While spontaneous Ca^2+^ signaling and electrical events can arise within the atrial chambers themselves [10], a clinically-recognised source of pro-arrhythmic signals are pulmonary vein sleeve cells (PVCs) that form sheaths surrounding the large pulmonary veins [11]. PVCs are present in all mammalian cardiovascular systems and utilise the same EC-coupling machinery as atrial and ventricular myocytes (described above). Even though PVCs are developmentally and anatomically distinct from atrial myocytes, both cell types are in electrical continuity [12]. Thus, PVCs are entrained by sinus rhythm because APs arising in the SA node would sweep across the atrial chambers, propagate out of the heart into the PVCs and cause them to contract. However, electrical mapping has demonstrated that ectopic activity can arise within PVCs [13], and propagate into the left atrial chamber. Moreover, ablation procedures that electrically isolate pulmonary veins from the posterior wall of the left atrium (the border of the two tissues) are highly successful in treating acute and sustained AF [14], [15], supporting the notion of PVC-initiated arrhythmias. The conditions leading to the generation of arrhythmic pacemaking sites within PVC sheaths is not understood, but is likely to involve the development of spontaneous Ca^2+^ signals [16].

The extent of the PVC sheath varies between animal species. Here, we took advantage of the situation in mice, where the PVCs extend from the left atrium to the pulmonary veins within the lung for several branching generations [17]. In previous work, we demonstrated the utility of lung slices for studying airway smooth muscle physiology [18]; lung slices provide an intact, multi-cellular preparation that retains *in situ* organizational and physiological characteristics of the lung, and are viable for many days. Because the PVCs are disconnected from the left atrial chamber, they no longer receive APs arising from the SA node. This electrical isolation allows the spontaneous Ca^2+^ signaling capacity of the PVCs to be evident without a background of SA node-evoked events. As required, the PVCs within a lung slice can be activated by application of electric field stimulation (EFS). In the present study, we characterised spontaneous and EFS-induced Ca^2+^ transients in PVCs to determine whether these two processes use similar signaling mechanisms. Moreover, we explored the hypothesis that the inherent spontaneous Ca^2+^ signals within PVCs could corrupt their electrical entrainment, and thereby lead to pro-arrhythmic outcomes.

## Methods

### Preparation of Lung Slices

For the preparation of lung slices [18] 8–12 week-old female BALB/C mice were killed by intraperitoneal Nembutal (pentobarbital sodium) injection, as approved by the Institutional Animal Care and Use Committee of the University of Massachusetts Medical School. After opening the chest cavity, the trachea was cannulated and lungs were inflated by injecting ∼1.0 ml of low melting point agarose (37°C, 1.8% in sHBSS; Life Technologies, Carlsbad, CA). Agarose was flushed out of the airways and into the alveoli by injection of 0.3 ml of air and the agarose was gelled by applying cold sHBSS to the lungs. The stiffened lungs were cut into ∼180 µm thick lung slices using a VF-300 microtome (Precisionary Instruments Inc., Greenville, NC). The presence of PVCs was confirmed by visual inspection at low magnification using the following criteria: the vein is not located next to an airway, spontaneous fibrillations and/or striations are visible in the cells surrounding the vein. Slices were kept in Dulbecco’s Modified Eagle Medium supplemented with antibiotics and antimycotics (Life Technologies) and 10% fetal bovine serum and kept in a humidified tissue culture incubator at 37°C with 10% CO_2_. Slices were used within 48 h of preparation, apart from experiments investigating the viability of PVCs. For imaging experiments, lung slices were mounted between 2 cover-glasses separated with silicon grease on a custom-built Perspex support, which allowed for a continuous superfusion of the lung slices with experimental solutions.

### Solutions and Compounds

Experiments were performed in sHBSS (KCl 5.3 mM, KH_2_PO_4_ 0.4 mM, NaCl 137.9 mM, NaH_2_PO_4_ 0.3 mM, NaHCO_3_4.2 mM, D-Glucose 5.6 mM, MgCl_2_ 0.5 mM, MgSO_4_ 0.4 mM, CaCl_2_ 1.3 mM, HEPES 20 mM), pH 7.4. Ca^2+^-free HBSS was made by supplementing nominally Ca^2+^-free sHBSS with 100 µM EGTA. Ryanodine and caged InsP_3_/PM (Propionoxymethylester) were purchased from Enzo Life Sciences (Farmingdale, NY). Unless otherwise mentioned, all other reagents were purchased from Sigma (St. Louis, MO).

### Imaging Experiments

Imaging experiments were performed on custom-built microscopes [19]. Video Savant software (IO Industries, CA) was used for recording images and controlling the timing of solution changes. Contractions were measured using a Nikon Diaphot 300 microscope with a 10x objective (N.A. 0.3) at 15, 30 or 120 images per second. Ca^2+^ imaging was performed on a custom-built 2-photon system (excitation wavelength of 800 nm), based on an Olympus IX71 microscope, using an oil immersion UApo 340 40x (N.A. 1.3) or PlanApo60x (N.A. 1.42) objective. Lung slices were loaded in the dark with 20 µM Oregon Green BAPTA-1 AM (Acetoxymethyl ester; Life Technologies) in presence of 0.1% Pluronic F127 (Life Technologies) and 200 µM sulfobromophthalein in sHBSS at 30°C for 1 hour, followed by a de-esterification period of at least 30 min at 30°C in the presence of sulfobromophthalein. Image acquisition was performed using Video Savant software at 30 images per second. Throughout the experiment, lung slices were perfused with sHBSS using a gravity-fed system. The solution exchange was controlled by Video Savant software. The 2-photon excitation wavelength was set by tuning the laser. The excitation light was reflected using a FF665-Di02-25×36 dichroic (Semrock, Rochester, NY), and the emitted fluorescence light was filtered using a short-pass barrier filter FF01-680/SP-25 (Semrock).

A custom-built confocal microscope, based on a Nikon Diaphot 200, with either a 40x Fluor (N.A. 1.3) or 63x Planapo (N.A. 1.4) oil-immersion objective was used for imaging immunofluorescence and di-8-ANNEPS (488 nm excitation for both). Specific staining of the PVC sarcolemma was achieved by incubation of lung slices with 25 µM di-8-ANNEPS (Life Technologies, Carlsbad, CA) for 30 min. The excitation (488 nm laser line) was reflected using a LPD01-488RU-25×36 dichroic (Semrock), and emitted fluorescence light was filtered using a long-pass LP02-488RU-25 filter (Semrock).

To avoid deterioration of the slices, or temperature gradients associated with superfusing cells, the experimental data shown in this study were obtained at room temperature (20°C). However, electrical pacing, contraction and spontaneous Ca^2+^ transients were evident at 20°C and 37°C (c.f. fig. S1 and *Results* section). The average frequencies of spontaneous Ca^2+^ transient activity were not significantly different at either temperature (2.3±0.2 and 2.5±0.2 Hz, 20°C and 37°C, respectively; see fig. S1C). Moreover, the effects of pharmacological reagents and experimental maneuvers (caffeine, ryanodine, KCl) were similar at both temperatures. A consequence of studying the slices at room temperature was that the electrical pacing rate was reduced from ∼8 Hz *in vivo* to ≤5 Hz. We speculate that the lack of effect of temperature on the frequency of spontaneous Ca^2+^ signals relates to the counterbalancing processes of Ca^2+^ release and reuptake being equally affected.

### Immunofluorescence

Immunostaining was performed in paraformaldehyde-fixed lung slices as previously described [20], but with the following modifications: 45 min fixation, 2 hour blocking in 10% BSA, 2 hour incubation of primary antibodies (RyR2 and RyR3, 1∶50 dilution in 0.1% triton containing PBS with 2% BSA) at 20°C. Alexa-Fluor 488 goat-anti-rabbit secondary antibody (Life Technologies) was incubated for 1 hour in the dark at 20°C (1∶500 dilution). Slices were mounted in Prolong Gold Mounting Medium (Life Technologies). Image analysis was performed with ImageJ [21].

### Electric Field Stimulation (EFS)

EFS (40 V, 10 ms duration) was applied using a Grass Instruments SD9 stimulator and platinum electrodes (0.005′ wire thickness, A–M systems), placed at the opposite ends of the imaging chamber. The EFS conditions were empirically determined by finding the minimal voltage condition that would reliably pace PVCs with no appreciable run-down of the responses.

### Statistics

Statistical analysis was performed in GraphPad Prism 6 (GraphPad software Inc., La Jolla, CA, USA), using a Mann-Whitney U-Test (2 datasets) or a Kruskall-Wallis Test followed by a Dunn’s post-hoc analysis (>2 datasets) to test for statistically significant differences by comparing the initial values the first 30 s in sHBSS at the start of the experiment to the values at the indicated time periods. P values <0.05 were considered significant, *P<0.05, **P<0.01, ***P<0.001. Data are shown as mean ± s.e.m.

## Results

### PVCs Respond to Electrical Pacing and Show Spontaneous Ca^2+^ Signals in the Absence of Stimulation

**Figure 1:**
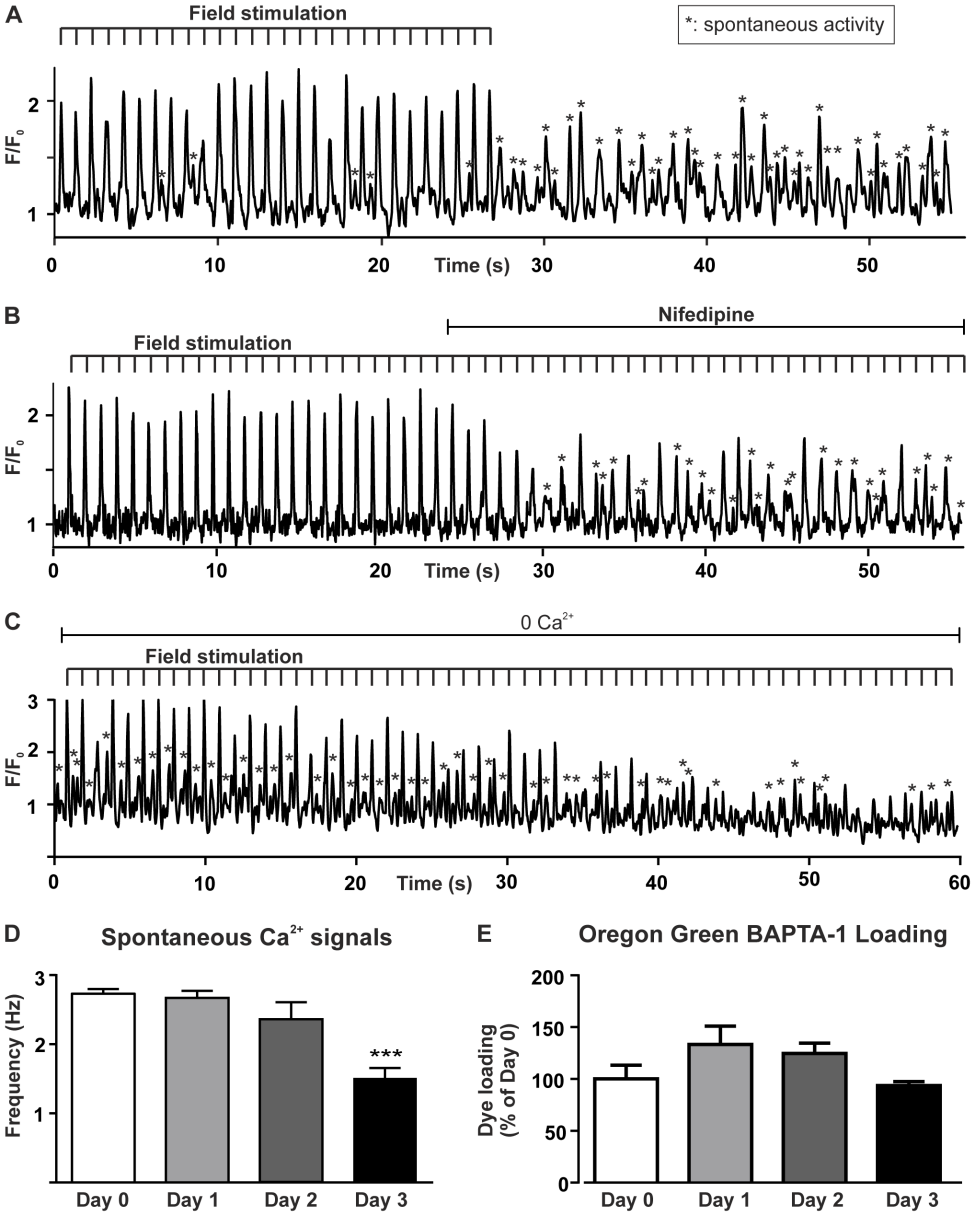
PVCs can be paced by electrical field stimulation (EFS), but are prone to showing spontaneous activity. (**A**) PVCs displayed rapid increases of cytosolic Ca^2+^ concentration in response to EFS. Between EFS pulses, some spontaneous Ca^2+^ activity was observed (indicated by asterisks). After termination of EFS, the cells continued to show spontaneous Ca^2+^ transients. (**B**) 100 µM nifedipine inhibited the ability of PVCs to respond to EFS pulses, causing the spontaneous Ca^2+^ transients to become prominent. (**C**) Removal of extracellular Ca^2+^ stopped responses to EFS, but spontaneous Ca^2+^ transients were still evident [See fig. 3 for an explanation how spontaneous and paced activity was distinguished]. (**D**) Quantification of the spontaneous Ca^2+^ transients on the day of the preparation (Day 0) and the three following days, illustrating that the spontaneous activity is not significantly different between day 0 and 2 (*n* = 7–36 cells, 2–4 slices). (**E**) Normalized intensity of Oregon Green BAPTA-1 in PVCs on the day of the preparation (Day 0) and the three following days (*n* = 2–19 cells, 1–10 slices).

To explore Ca^2+^ signaling mechanisms within PVCs, lung slices were loaded with the Ca^2+^-sensitive indicator Oregon Green BAPTA-1 and examined with real-time 2-photon microscopy. PVCs displayed rapid, transient Ca^2+^ increases in response to electric field stimulation (EFS; Fig. 1). Most PVCs followed such pacing for long periods with consistent responses (*n* >50 slices; maximum observation time 17 minutes). Disruption of electrical stimulation by stopping EFS (Fig. 1A), application of the VOCC inhibitor nifedipine (100 µM; Fig. 1B) or removal of extracellular Ca^2+^ (Fig. 1C) inhibited the pacing-induced Ca^2+^ responses and provoked the occurrence of spontaneous Ca^2+^ transients. These data illustrate two key aspects of PVC Ca^2+^ signaling: the regular Ca^2+^ signals underpinning physiological EC-coupling, and pro-arrhythmic, spontaneous Ca^2+^ transients.

We were mindful that damage to PVCs during preparation of lung slices could induce the spontaneous Ca^2+^ signalling that we observed. However, the slices remained viable, with no obvious visual indication of slice deterioration, in culture for 3 days. The average frequencies of spontaneous Ca^2+^ transients were not statistically different up to 2 days after preparing the lung slices (Fig. 1D). In addition, PVCs could be electrically paced by EFS for 2 days after slice preparation (later days were not examined). PVCs consistently loaded with the Ca^2+^-sensitive indicator to the same level for 3 days post preparation (Fig. 1E). Moreover, the Ca^2+^ indicator was stably retained within PVCs up to 3 days post preparation. Active PVCs did not label with propidium iodide (PI; fig. S2). These data indicate that the PVCs were vital and not damaged during slice preparation, and that they did not rapidly de-differentiate in culture. To ensure consistency, all data in the present study were obtained using slices within 48 hours after preparation. Atrial myocytes within slices of the right atrial chamber, prepared using the same buffer solutions and conditions as for PVCs, could also be paced using EFS, but did not show prominent spontaneous Ca^2+^ transients (fig. S3). These observations indicate that PVCs and atrial myocytes have a different propensity for spontaneous Ca^2+^ signaling.

### PVC Organization, Morphology and Contraction

**Figure 2:**
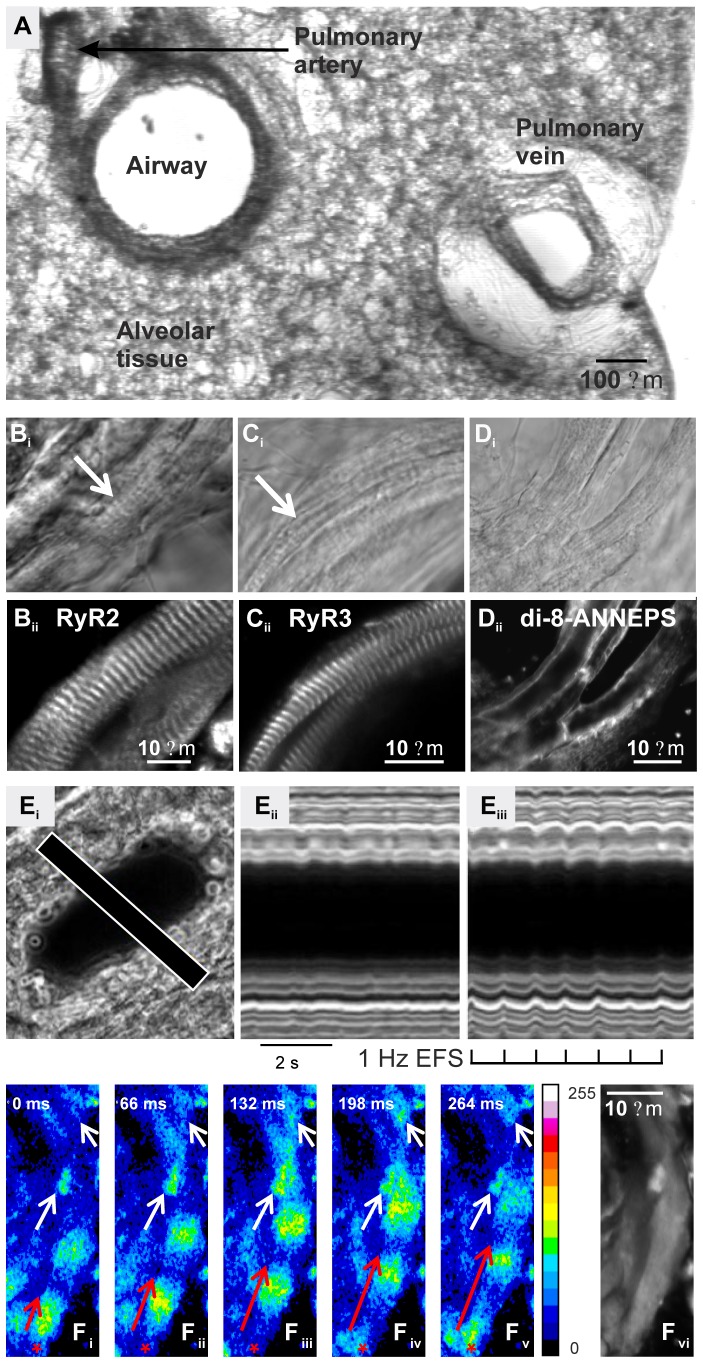
Characterization of PVCs and their spontaneous Ca^2+^ signaling in mouse lung slices. (**A**) Cross-section of a lung slice showing a lone pulmonary vein, and the relative position of an airway and its associated pulmonary artery within the alveolar tissue. (**B_i_–C_ii_**) Phase-contrast images of PVCs revealed the characteristic striated pattern (arrows) associated with sarcomeres of cardiac myocytes that (**B_ii_**) overlap with the striated expression pattern of RyR2 and (**C_ii_**) RyR3 detected by immunofluorescence. (**D_i_**) Light microscopic image and (**D_ii_**) membrane staining of PVCs with di-8-ANNEPS predominantly highlighted peripheral membranes with little evidence for internal transverse-tubules (*n* = 38 cells, 5 slices). (**E_i_**) Differential interference contrast image of a pulmonary vein cross section that displayed (**E_ii_**) small, non-coordinated spontaneous contractions and (**E_iii_**) larger, coordinated contractions in response to EFS (1 Hz, black tick marks; line-scan analysis along line indicated in E_i_). (**F_i–v_**) Spontaneous Ca^2+^ increases that either remain localized and only spread over a limited area (white arrows) or that travel as a Ca^2+^ wave through individual cells (red arrow). The Ca^2+^ waves often originated in the same location (e.g. red asterisks) and spread in the same direction with each wave. Relative fluorescence (Ca^2+^) increases are indicated by the pseudocolor bar (F/F_0_). The time interval between images is indicated in each panel. (**F_vi_**) Grey-scale fluorescence image of the PVCs.

Pulmonary veins with a PVC myocardial sheath are found in mouse lung slices cut from the hilus region, where the pulmonary veins exit the lungs, to a distance approximately half-way through the lung lobe towards the periphery of the lungs (Fig. 2A). The myocardial sheath is absent in more peripheral lung slices. The pulmonary veins (also known as intrapulmonary veins within the lung) are easily identified by their lone appearance within the lung alveoli tissue, as compared to the consistently paired association of a pulmonary artery with an airway (Fig. 2A). The PVCs surrounding each vein could be identified using bright-field or phase-contrast optics as relatively large cells, which often displayed regular cellular striations that are typical of myocytes (Figs. 2B_i_–D_i_). Immunostaining for the cardiac isoforms of RyR (types 2 and 3) revealed a striated distribution of these Ca^2+^ channels (Figs. 2B_ii_ and C_ii_, respectively) within the PVCs. These striations were 1.8±0.1 µm apart (*n = *5 regions per slice, 6 slices), similar to the RyR (z-line) distribution in cardiac myocytes [22]. The membrane dye di-8-ANNEPS predominantly stained only the periphery of PVCs in >98% of cells, indicating that PVCs do not express an extensive transverse-tubule system (Figs. 2D_i_ and D_ii_). Collectively, these data suggest that PVCs structurally resemble rodent atrial myocytes [22] [23].

### Contraction of Pulmonary Veins

In addition to their location and morphology, PVCs surrounding pulmonary veins could be readily identified by their prominent spontaneous contractions (Fig. 2E). The nature of this contractile activity was heterogeneous. In some veins, repetitive ripples of contraction propagated asynchronously along individual PVCs (frequency range 0.6–1.3 Hz). In other veins, multiple PVCs displayed more rapid, uncoordinated contractile activity. The numbers of PVCs involved ranged from a limited sub-population of cells, to all the PVCs surrounding the vein.

In many lung slices, asynchronous spontaneous PVC contractile activity could be coordinated into synchronous contractions by EFS, with a consequent reduction in the lumen size of the pulmonary vein during each contraction (Figs. 2E_i–iii_; Video S1). During spontaneous contractions, the relative reduction in pulmonary vein lumen size was 0.2±0.1%, which increased significantly to 6.3±0.7% (P<0.01) during the EFS-induced contraction (*n* = 6 slices). Synchronised contraction only occurred during EFS; spontaneous contractile activity resumed with the termination of EFS.

### Spontaneous Ca^2+^ Signaling within PVCs and Entrainment by EFS

The overwhelming majority of PVCs displayed spontaneous Ca^2+^ activity in the absence of EFS (*n* >250 slices). The spontaneous Ca^2+^ transients were heterogeneous, and ranged from localized Ca^2+^ increases (Ca^2+^ sparks) to Ca^2+^ waves that propagated along single PVCs (Figs. 2F; Video S2 and Video S3). The frequency of this spontaneous activity varied from cell to cell within a slice (range 0.3 to 3.4 Hz; mean 2.0±0.2 Hz; *n* = 24 cells, 5 slices). Ca^2+^ wave propagation occurred with a speed of 70.2±3.5 µm/s, with a mean wave duration of 388.2±18.8 ms (*n = *32 waves, 20 slices). Spontaneous Ca^2+^ signaling could be imaged within individual PVCs for >15 minutes with little change in activity.

**Figure 3:**
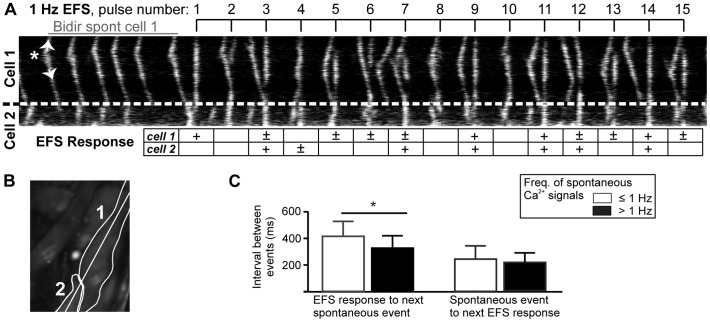
Effect of Electrical Field Stimulation (EFS) on PVC Ca^2+^ signals. (**A**) Line-scan analysis of spontaneous Ca^2+^ signals and responses to 1 Hz EFS in two neighboring cells. The cells are outlined in the 2-photon fluorescence image of PVCs in a lung slice shown in (**B**). In (**A**), the cell border is depicted by the dashed line. The spontaneous Ca^2+^ activity in Cell 1 shows a bidirectional wave (origin indicated by an asterisks, arrowheads indicate direction). The timing of the Ca^2+^ increases in both cells is independent of each other, but did occasionally coincide. EFS caused whole-cell Ca^2+^ increases, which are shown as vertical straight lines. EFS timing and pulse numbers are indicated by the top bar. The ability of Cell 1 or 2 to respond to EFS is indicated in the table; ‘+’ indicates full response, while ‘±’ indicates an incomplete response. (**C**) Summary, showing that the latency from an EFS-induced Ca^2+^ signal to the next spontaneous Ca^2+^ transient is significantly longer for PVCs with infrequent spontaneous Ca^2+^ activity (left). In contrast, the latency from a spontaneous Ca^2+^ transient to the next EFS-induced Ca^2+^ signal did not depend on the frequency of the spontaneous activity (right) (*n* >166 events in 10 cells, 3 slices).

Typical spontaneous Ca^2+^ signals are illustrated by the line-scan plot in fig. 3A. This plot shows Ca^2+^-dependent fluorescence within a pair of PVCs (the 8-pixel-wide continuous line that was sampled is shown in fig. 3B). Within such line-scan plots, distance across the cells is represented vertically while time progresses horizontally. Propagating Ca^2+^ waves are evident as diagonal lines. Spontaneous Ca^2+^ waves can be seen to originate in the centre of cell 1 (asterisk), and propagate bi-directionally to the poles of the cell (arrowheads). The Ca^2+^ waves stopped at the boundary between cells 1 and 2 (dashed line). Cell 2 had its own intrinsic rhythm, and displayed Ca^2+^ waves that sometimes temporally coincided with those of cell 1.

EFS-evoked Ca^2+^ transients are evident in fig. 3A as vertical lines that coincide with EFS pulses. EFS restored the effect of sinus rhythm by evoking Ca^2+^ signals (and triggering contraction) within the PVCs. However, the efficacy of each EFS pulse varied on a pulse-to-pulse basis, and ranged from inducing a whole-cell Ca^2+^ signal to having no effect. For example, in fig. 3A, only on 3 occasions did EFS pulses cause a whole-cell Ca^2+^ signal simultaneously in both cells (at pulses 9, 11, 14; both Cell 1 and 2 are marked ‘+’). The other EFS pulses correlated with one of three types of responses; i) only one cell showed a whole-cell response (pulse 1, Cell 1 is marked ‘+’), ii) one or both cells showed an incomplete response (pulses 3–7, 12, 13 and 15, marked ‘±’) or iii) neither cell responded (pulses 2, 8 and 10). A plausible explanation for variable effects of EFS is that the PVCs enter a refractory period after a Ca^2+^ rise during which they cannot respond to another Ca^2+^-releasing stimulus. Therefore, if a cell responds to EFS with a whole-cell response, the subsequent spontaneous Ca^2+^ signal is inhibited or delayed. Conversely, if a spontaneous Ca^2+^ wave occurs just prior to an EFS pulse, the cell cannot respond with a Ca^2+^ transient. In essence, Ca^2+^ signals evoked by EFS delayed the timing of spontaneous Ca^2+^ activity and *vice versa*.

We found that the average latency between an EFS-evoked Ca^2+^ signal and a subsequent spontaneous Ca^2+^ event was correlated with the frequency of the intrinsic spontaneous Ca^2+^ activity. The latency was longer in cells with a sparse spontaneous Ca^2+^ activity (<1 Hz) as compared to cells with more frequent spontaneous Ca^2+^ activity (>1 Hz, fig. 3C, left). An explanation for these observations is that cells with higher frequencies of spontaneous Ca^2+^ signals have a greater capacity for restoring SR Ca^2+^ content. This would enable PVCs to recover more quickly from an EFS-induced Ca^2+^ pulse, as well as predispose the PVCs to show more frequent spontaneous Ca^2+^ signals. Interestingly, there was no similar relationship governing the interval between a spontaneous Ca^2+^ signal and a subsequent Ca^2+^ pulse induced by EFS (Fig. 3C, right). This is likely due to the fact that an EFS pulse triggers a Ca^2+^ influx signal to promote internal Ca^2+^ release. The EFS-induced Ca^2+^ signals may therefore be less dependent on SR Ca^2+^ refilling in comparison to the spontaneous Ca^2+^ events.

**Figure 4:**
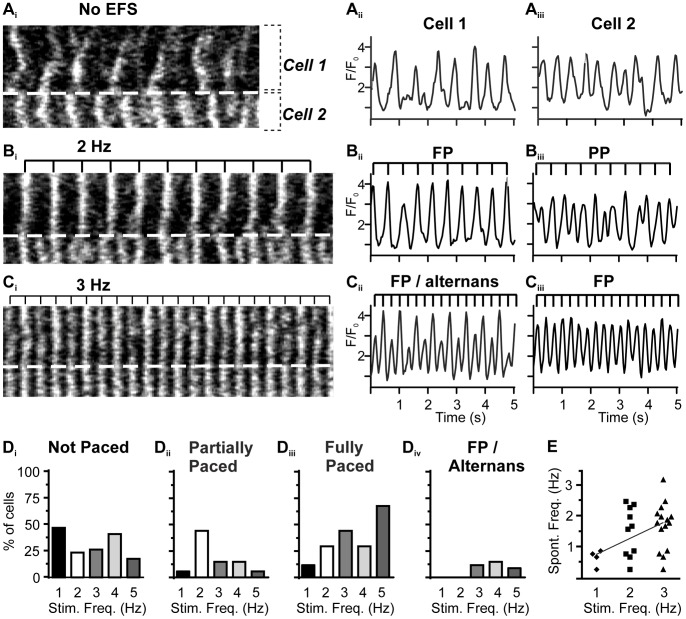
Heterogeneity in the pulsed responses of neighboring PVCs to increasing frequencies of EFS. At each EFS frequency (0 to 3 Hz), the responses of 2 contiguous PVCs are illustrated with a line-scan analysis (left, **A_i_** to **C_i_**) and a fluorescence (Ca^2+^) trace for Cell 1 (**A_ii_** to **C_ii_**) and Cell 2 (**A_iii_** to **C_iii_**). Within the line-scan image, the cell border between the PVCs is indicated by the dashed line. The timing of the EFS pulses is indicated by the top bar. (**A**) PVCs displayed spontaneous Ca^2+^ activity in the absence of EFS. (**B**) With 2 Hz EFS, Cell 1 displayed full pacing (FP) while Cell 2 showed partial pacing (PP). (**C**) With 3 Hz EFS, Cell 1 displayed alternans while Cell 2 was fully paced. (**D_i–iv_**) Summary, showing the predominate forms of response induced by different EFS frequencies (*n* = 25 cells, 4 slices). (**E**) Correlation of spontaneous Ca^2+^ transient frequency and the EFS stimulation frequency required for full pacing (*n* = 25 cells, 4 slices).

We hypothesised that increased EFS frequency might overcome the confounding effects of spontaneous Ca^2+^ signals and provide a more reliable entrainment of PVCs. However, elevated EFS frequencies only partially improved entrainment, and moreover caused markedly heterogeneous cell responses. For example, fig. 4 shows the responses of two adjacent PVCs to EFS applied at 2 and 3 Hz. Prior to EFS, both cells displayed independent spontaneous Ca^2+^ signals (Fig. 4A). Cell 1 was fully paced (without any remaining spontaneous activity, FP) with 2 Hz (Fig. 4B_ii_) and 3 Hz EFS. However, in response to 3 Hz, Cell 1 displayed ‘alternans’ (alternating large and small Ca^2+^ transients, fig. 4C_ii_). Conversely, Cell 2 was only partially paced (PP) with 2 Hz EFS (Fig. 4B_iii_), but was fully paced (FP) with 3 Hz EFS without alternans (Fig. 4C_iii_). These observations indicate that while increasing the frequency of EFS could sometimes entrain spontaneous Ca^2+^ signals, other forms of pro-arrhythmic activity, e.g. alternans, could ensue.

Figure 4D quantitatively summarizes the relationship between the frequency of EFS and the pattern of PVC Ca^2+^ signaling. It is evident that PVCs were more successfully paced as the EFS frequency increased. However, not all PVCs were paced (Fig. 4D_i_). PVCs that were partially paced were more evident at lower EFS frequencies (Fig. 4 D_ii_), whilst higher EFS frequencies were most effective for complete entrainment (Fig. 4D_iii_). However, the higher EFS frequencies were also capable of generating alternans (Fig. 4D_iv_). We suggest that the heterogeneity of EFS-induced Ca^2+^ signaling arises from the relative timing of an EFS pulse with respect to the intrinsic spontaneous Ca^2+^ signaling activity; it is easier to fully pace cells which show little spontaneous activity, and more difficult to pace cells with a high frequency of spontaneous activity. In support of this conclusion, we observed that the minimal EFS frequency required for fully paced cells correlated with the intrinsic frequency of spontaneous Ca^2+^ signals (Fig. 4E). Those cells with more frequent spontaneous Ca^2+^ signals required higher EFS frequencies. Moreover, at all EFS frequencies tested, the cells that could be fully paced had a lower average frequency of spontaneous Ca^2+^ signals compared to those cells that could not be fully paced. With 1 Hz EFS, for example, fully paced PVCs had an average spontaneous Ca^2+^ signal frequency of 0.7±0.1 Hz, whereas those cells that could not be fully paced by 1 Hz EFS had an average spontaneous Ca^2+^ signal frequency of 1.9±0.2 Hz (*n* = 20, 5 slices). We suggest that all PVCs have unique periodicities of spontaneous Ca^2+^ signaling, and this determines the cells’ refractory periods. In some cells, the frequency of spontaneous Ca^2+^ signaling is so high that EFS is ineffective. It should be noted that whilst there is a clear impact of the frequency of spontaneous Ca^2+^ signals on the pacing of PVCs, this is not the only factor. We also observed cells with similar frequencies of spontaneous Ca^2+^ signaling responding differently to the same EFS pulse. This implies heterogeneity in the responsiveness of the cells to EFS in addition to the effects of spontaneous Ca^2+^ signaling.

### Ca^2+^ Re-addition Induces Rapid Ca^2+^ Waves within PVCs and Prevents Electrical Synchronisation

**Figure 5:**
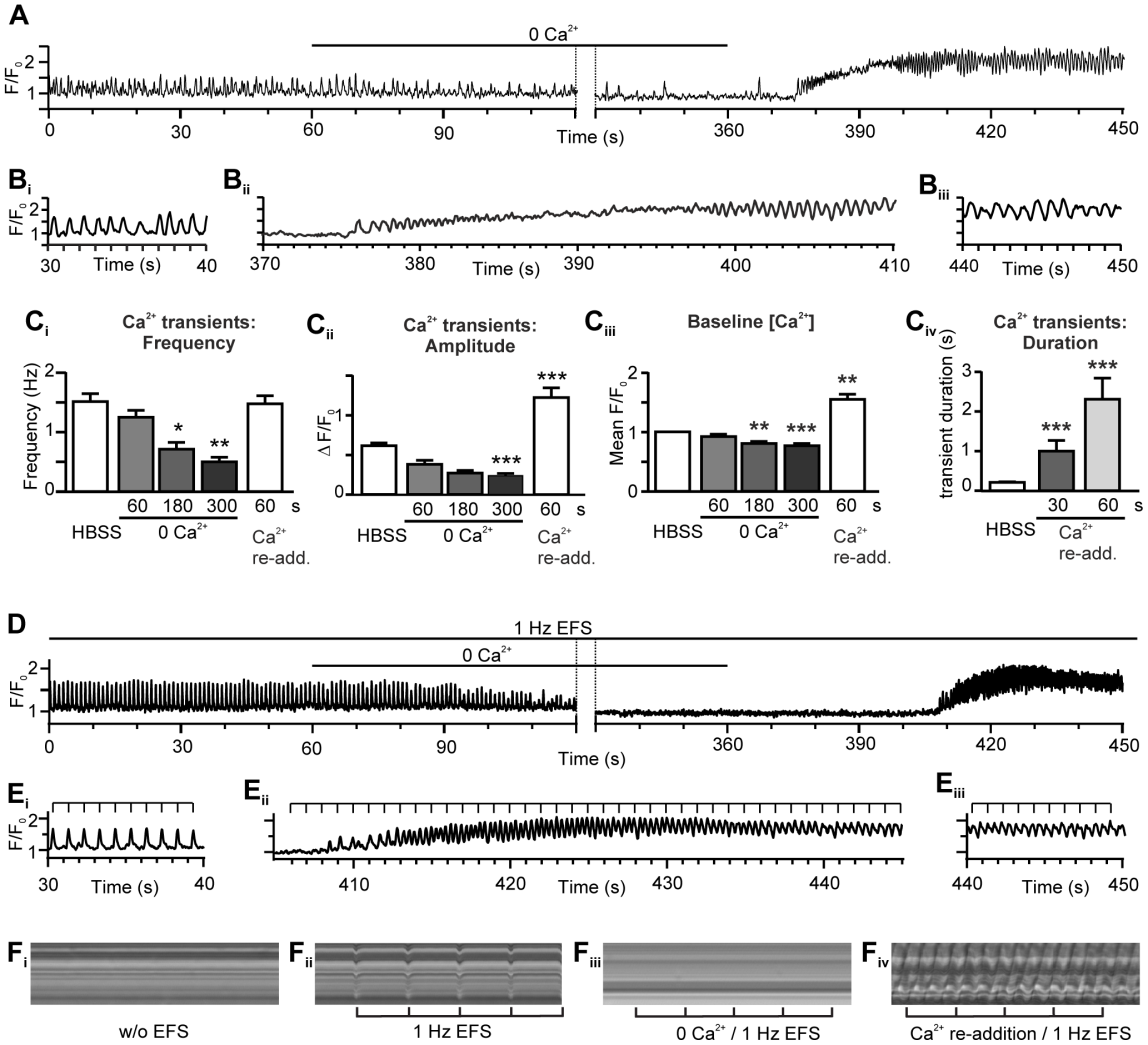
Response of PVCs to the removal and re-addition of external Ca^2+^. (**A**) A fluorescence (Ca^2+^) trace showing the reduction of spontaneous Ca^2+^ transients during a period of extracellular Ca^2+^ removal, and increased spontaneous Ca^2+^ transient activity after Ca^2+^ re-addition. (**B**) A representative example of (**B_i_**) the spontaneous Ca^2+^ activity before Ca^2+^ removal, (**B_ii_**) the restoration of the Ca^2+^ transients during Ca^2+^ re-addition and (**B_iii_**) the prolonged Ca^2+^ waves after Ca^2+^ re-addition. (**C_i–iv_**) Quantification of the Ca^2+^ responses of PVCs to Ca^2+^ removal and re-addition. (**C_i_**) The frequency of the spontaneous Ca^2+^ transients is reduced by Ca^2+^ removal. (**C_ii_**) The amplitude (ΔF/F_0_) of the spontaneous Ca^2+^ transients and (**C_iii_**) the cytosolic Ca^2+^ concentration transients are both reduced by Ca^2+^ removal and increased after Ca^2+^ re-addition. (**C_iv_**) The duration of Ca^2+^ transients increases at Ca^2+^ re-addition (*n* = 35 cells, 12 slices). (**D**) A fluorescence (Ca^2+^) trace showing the effect of external Ca^2+^ removal while 1 Hz EFS was applied. EFS-induced pacing of the cell was lost during Ca^2+^ removal and was not re-established when extracellular Ca^2+^ was restored. (**E**) Summary of the (**E_i_**) Ca^2+^ responses to EFS before the Ca^2+^ removal, (**E_ii_**) Ca^2+^ signals following Ca^2+^ re-addition and (**E_iii_**) the prolonged Ca^2+^ waves established after Ca^2+^ re-addition. (**F**) shows (**F_i_–_iv_**) Line-scan analysis of phase-contrast images measuring pulmonary vein contraction (*cf*. Fig. 1). (**F_i_**) No contraction without EFS. (**F_ii_**) Coordinated contractions with 1 Hz EFS. (**F_iii_**) In the absence of external Ca^2+^, EFS did not evoke contractions. (**F_iv_**) Strong uncoordinated contractions after the re-addition of Ca^2+^.

Superfusion of non-paced PVCs with Ca^2+^-free medium caused a progressive reduction in the amplitude and frequency of spontaneous Ca^2+^ signals (Fig. 5A), and of the basal cytosolic Ca^2+^ concentration, until the PVCs became quiescent (quantified in figs. 5F_i–iv_). In 58% of the PVCs, the spontaneous Ca^2+^ activity was fully inhibited within 5 min of Ca^2+^ removal (mean time to inhibition: 209.5±15.3 s). This inhibition of the spontaneous Ca^2+^ signals also correlated with the loss of spontaneous PVC contractions. These data indicate i) that spontaneous Ca^2+^ signals arise via Ca^2+^ release from intracellular stores, ii) that PVCs have a substantial capacity for recycling Ca^2+^, and iii) that PVCs require Ca^2+^ influx to sustain spontaneous Ca^2+^ activity and contraction.

The re-addition of extracellular Ca^2+^ (1.3 mM) did not restore the initial pattern of spontaneous Ca^2+^ activity, but rather induced an entirely different form of Ca^2+^ signaling. Within 40 to 60 s of Ca^2+^ re-addition, PVCs began to display Ca^2+^ waves that progressively increased in amplitude, frequency and spatial propagation (Figs. 5A, B_ii_; quantified in figs. 5C_i–iv_). Moreover, these Ca^2+^ waves had a longer duration than the original spontaneous activity (compare Figs. 5B_iii_ and B_i_; fig. 5C_iv_). Consequently, the cytosolic Ca^2+^ concentration did not have time to recover between wave fronts, so the diastolic Ca^2+^ level became elevated. Typically, all the PVCs surrounding a pulmonary vein displayed Ca^2+^ waves in response to re-addition of extracellular Ca^2+^ (100% of slices; *n* = 12 slices). The Ca^2+^ waves were associated with strong PVC contractions, but the contractions were not coordinated between cells with the result that the layer of PVCs fibrillated asynchronously. Significantly, the rapid Ca^2+^ waves and strong contractions persisted for some time after Ca^2+^ re-addition, and did not return to the original pattern of spontaneous activity within 15 minutes. The spontaneous Ca^2+^ waves in PVCs under control conditions were predominantly *intracellular*. The Ca^2+^ removal/re-addition experiments presented a situation in which intracellular Ca^2+^ waves frequently turned into *intercellular* Ca^2+^ waves (an example is shown in Fig. S4).

Application of EFS either prior to, or during, Ca^2+^ re-addition did not prevent the PVCs from developing long-lasting Ca^2+^ waves (Fig. 5D and E; compare spontaneous Ca^2+^ transients in Fig. 5E_i_ with the increased duration Ca^2+^ waves in Fig. 5E_iii_; Video S3). Even slices showing an initial high degree of entrainment to EFS (Fig. 5 E_i_) were driven to the fibrillated state that was unresponsive to EFS (Figs. 5 E_ii_ and E_iii_) by removal and re-addition of extracellular Ca^2+^. Similarly, EFS could not re-synchronize the contractile activity of the PVCs following re-addition of extracellular Ca^2+^ (Figs. 5 F_i–iv_). Re-addition of a lower extracellular Ca^2+^ concentration (500 µM instead of 1.3 mM), slowed the onset of the rapid Ca^2+^ waves and contractions, but the PVCs eventually reached a similar state of fibrillation that was unresponsive to EFS.

Our data indicate that PVCs can progress to an unrecoverable, EFS-insensitive form of Ca^2+^ signaling that is characterized by Ca^2+^ waves with a longer duration and a persistent elevation of cytosolic Ca^2+^. Moreover, other maneuvers (see below) that promoted Ca^2+^ influx or altered Ca^2+^ homeostasis could also cause PVCs to display a rapid, EFS-resistant, form of Ca^2+^ signaling.

**Figure 6:**
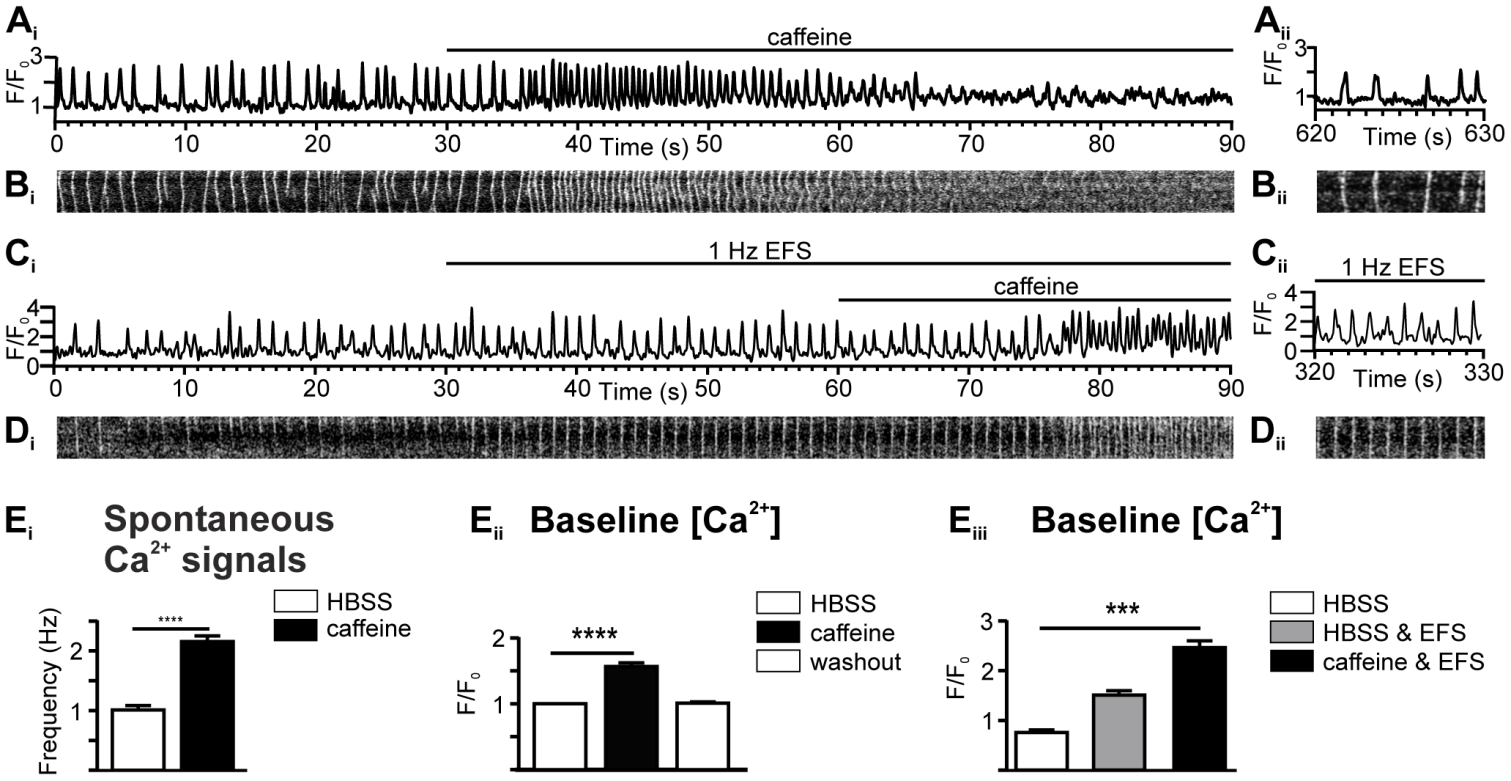
Caffeine stimulates Ca^2+^ signals in PVCs. (**A_i_**) A Ca^2+^ trace and (**B_i_**) line-scan analysis of spontaneous Ca^2+^ signals, illustrating that 1 mM caffeine activated a reversible increase in the spontaneous Ca^2+^ activity, which was reversible (**A_ii_** and **B_ii_**). **(C_i_)** A Ca^2+^ trace and (**D_i_**) line-scan analysis of Ca^2+^ signals paced by 1 Hz EFS illustrating that 1 mM caffeine increased the spontaneous Ca^2+^ activity and caused a loss of pacing, which was reversible (**C_ii_** and **D_ii_**). (**E**) Summary of the response to 1 mM caffeine showing (**E_i_**) increased frequency of the spontaneous Ca^2+^ signals and increased basal Ca^2+^ levels in (**E_ii_**) the absence of EFS (*n* = 46 cells, 9 slices) or (**E_iii_**) the presence of EFS (*n* = 35 cells, 8 slices).

The fact that spontaneous Ca^2+^ signals continue for some time in the absence of extracellular Ca^2+^ suggests that these Ca^2+^ signals depend on Ca^2+^ release from internal stores. Consistent with this idea, we observed that caffeine (a RyR agonist; 1 mM) increased the frequency of spontaneous Ca^2+^ transients and typically caused a sustained elevation of cytosolic Ca^2+^ (Fig. 6A and B, quantified in E_i_ and E_ii_). PVCs with EFS showed a similar caffeine-evoked increase in spontaneous Ca^2+^ activity, and consequent loss of entrainment in 47% of the cells (Fig. 6C and D, quantified in E_iii_). The effects of caffeine were reversible within 30–60 s (Fig. 6A_ii_ and B_ii_), and the PVCs could be re-entrained by EFS (compare responses between 30 to 60 seconds in Fig. 6 C_i_/D_i_ with C_ii_/D_ii_). Ryanodine (a RyR antagonist; 10 µM) inhibited the spontaneous Ca^2+^ signals within ∼150 s (Figs. S5A and B) and completely blocked EFS-evoked Ca^2+^ signals within ∼100 s in an irreversible manner (Figs. S5C and D). We attempted to use a maximal caffeine concentration to provoke emptying of the SR Ca^2+^ store. This is a commonly-employed method for assessing the SR Ca^2+^ load in cardiomyocytes. However, we found that 20 mM caffeine did not produce the same large, monophasic Ca^2+^ responses in PVCs that it evokes in isolated cells. Rather, application of 20 mM caffeine caused an acceleration of Ca2+ transients, leading to a persistent increase of the cytosolic Ca^2+^ concentration (Fig. S6).

Inositol 1,4,5-trisphosphate receptors (InsP_3_R) did not appear to play a role in spontaneous Ca^2+^ responses. Photolytic release of InsP_3_ did not increase the cytosolic Ca^2+^ concentration in PVCs (mean F/F_0_ 1.0±0.01 before and 1.1±0.03 after uncaging, *n* = 23 cells, 8 slices). In contrast, airway smooth muscle cells within the same slices showed substantial Ca^2+^ signals following photolysis of caged InsP_3_ (mean F/F_0_ 1.1±0.01 before and 1.7±0.01 after uncaging, *n* = 3 cells, 2 slices). Furthermore, 2-aminoethoxydiphenyl borate (2-APB; an InsP_3_R antagonist; 5 µM), did not alter the frequency or spatial properties of spontaneous Ca^2+^ signaling within PVCs (initial frequency in absence of 2-APB: 1.4±0.16 Hz, 180 s after 2-APB 1.3±0.19 Hz, 300 s after 2-APB 1.5±2.3 Hz,; n.s.; *n* = 15 cells, 4 slices).

**Figure 7:**
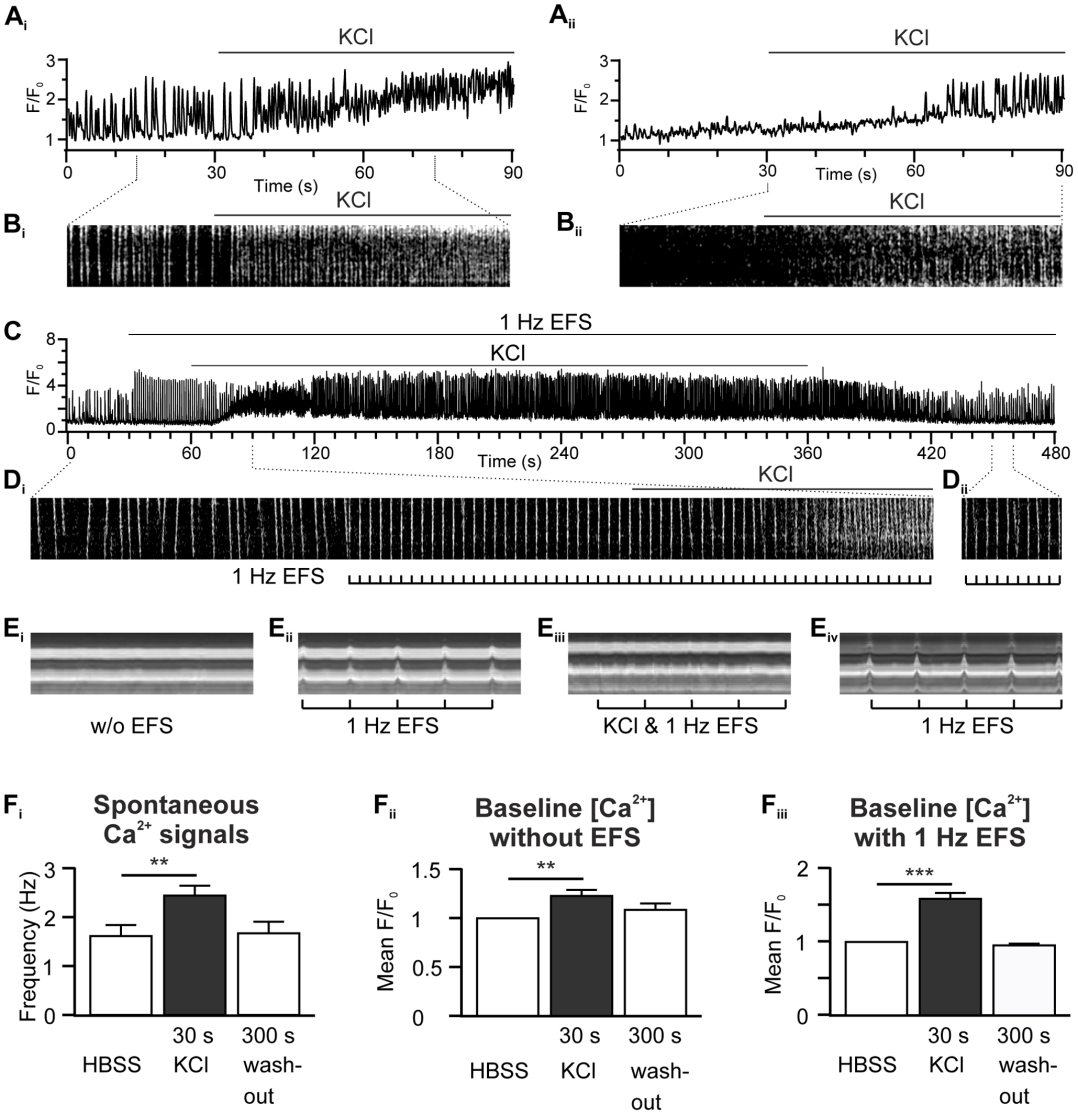
KCl stimulates Ca^2+^ signals in PVCs. (**A_i_, A_ii_**) Ca^2+^ traces and (**B_i_, B_ii_**) line-scan analysis (selected details) showing that KCl increased spontaneous Ca^2+^ signals in PVCs. (**C**) Ca^2+^ trace and (**D_i_, D_ii_**) selected line-scan analysis showing the that KCl reversibly caused a loss of pacing and increased spontaneous Ca^2+^ signals in PVCs during 1 Hz EFS. (**E_i_** –**_iv_**) Line-scan analysis of phase-contrast images of a pulmonary vein measuring contraction (cf. Fig. 1). (**E_i_**) No contractions without EFS. (**E_ii_**) 1 Hz EFS induced contractions. (**E_iii_**) KCl changed the coordinated contractions to uncoordinated fibrillations. (**E_iv_**) EFS responses were restored after KCl wash-out. (**F_i–iii_**) Quantification of the PVC responses to KCl addition. Summaries showing that KCl (**F_i_**) increased the frequency of the spontaneous Ca^2+^ signals and (**F_ii_**) the basal Ca^2+^ concentration in PVCs without EFS (*n* = 17 cells, 4 slices). KCl also (**F_iii_**) increased the basal Ca^2+^ concentration in PVCs paced with 1 Hz EFS (*n* = 24 cells, 4 slices).

Depolarising PVCs by elevating the extracellular KCl concentration (from 5.3 mM to 50 mM) progressively increased the frequency of spontaneous Ca^2+^ signals (Figs. 7A_i_ and B_i_, quantified in fig. 7F_i_) and stimulated the occurrence of spontaneous events in PVCs that were previously silent (Figs. 7A_ii_ and B_ii_). KCl treatment had a similar effect to that of caffeine and induced rapid Ca^2+^ waves superimposed on an elevated cytosolic Ca^2+^ level, which were reversible upon washout (Fig. 7F_ii_). The effects of KCl on PVCs during EFS were similar; KCl induced the progressive development of rapid Ca^2+^ waves on an elevated Ca^2+^ baseline to the point that synchronised Ca^2+^ signals and contractions induced by EFS were prevented (Figs. 7C and D, F_iii_; contractions in figs. 7E_i–iv_). Pacing was lost after 19±1.2 s of KCl application. As with caffeine, the rapid Ca^2+^ waves evoked by KCl could be reversed upon washout and entrainment re-established 67.1±3.6 s after washout (Fig. 7D_ii_).

Nifedipine (100 µM) stopped EFS-induced pacing within 63±26.1 s (Figs. S7A and B) and contraction (Fig. S7C). While inhibiting the EFS-induced Ca^2+^ signals, nifedipine promoted the re-occurrence of spontaneous Ca^2+^ events which had been previously suppressed by EFS (Figs. 1B and S7B_i_–B_iv_). In the absence of EFS, nifedipine reduced the frequency of spontaneous Ca^2+^ signals in a reversible manner (Figs. S7D and E). This latter effect indicates that VOCCs provide a source of Ca^2+^ influx to support or trigger spontaneous Ca^2+^ events. Verapamil (another L-type VOCC blocker; 100 µM) had similar effects to nifedipine (data not shown).

## Discussion

In the present study, we characterised spontaneous and EFS-induced Ca^2+^ signals within PVCs, and examined the effect of spontaneous Ca^2+^ signaling on the ability of the PVCs to respond to sustained electrical pacing. The use of the lung slice preparation for examining PVCs has the advantages of easy preparation, a cellular architecture resembling the *in situ* organization, compatibility with microscopy and a viability lasting several days. Spontaneous Ca^2+^ signals have previously been recorded in PVCs from various species [24]–[26], but their influence on pacing has not been resolved. A critical aspect of PVCs that was apparent in this study was the heterogeneity between cells in terms of Ca^2+^ handling. PVCs display repetitive spontaneous Ca^2+^ release events, but the frequencies and spatial properties of those events are unique for each cell. Correspondingly, PVCs show heterogeneous responses to electrical pacing such that neighboring cells can become, and remain, desynchronised. Such heterogeneity is known to serve as a basis for cardiac arrhythmias, and this may underlie the generation of arrhythmic events within pulmonary veins.

PVCs play an important role in the genesis and maintenance of AF [15], [27]–[29]. Electrical mapping studies have pinpointed PVCs as a source of phase 4 depolarisations (i.e. APs during the normally quiescent diastolic period) [30] that can pervade the atria via the junction of the atrium and PV at the left atrial antrum. Ablation procedures that prevent the electrical continuity between PVCs and atrial myocytes are highly effective in treating AF, but can have serious side-effects [31]. Despite the confirmation of PVCs as a source of ectopic activity, the events that occur within these cells to generate arrhythmic signals are less clear [11]. With respect to the mechanisms generating arrhythmias, numerous studies, using a range of cardiac cell types, have demonstrated that spurious Ca^2+^ signals are a potent source of arrhythmic activity [32]. Moreover, 3-dimensional electrical mapping of pulmonary veins identified spontaneous activity occurring as discrete focal events, consistent with the notion that cellular Ca^2+^ signals may be involved [33].

Under normal conditions, contractile myocytes and conductive cells within the heart experience a Ca^2+^ signal during the passage of each AP. At the end of an AP, cytosolic Ca^2+^ must recover to diastolic levels in order for the cells to reset and be fully responsive to the next stimulation. Typically, there is a diastolic period of hundred(s) of milliseconds between each AP where Ca^2+^ remains at the resting level of ∼100 nM. Consequently, spontaneous Ca^2+^ signals may corrupt this normal activity and recovery cycle of cardiac cells in a number of ways [32]. For example, Ca^2+^ causes the activation of electrogenic ion transporters, such as Na^+^/Ca^2+^ exchange or Ca^2+^-dependent chloride flux that leads to cellular depolarisation. Ca^2+^ signals occurring during the recovery of an AP, or in the normally quiescent diastolic period, give rise to ‘triggered activity’ such as early- or delayed-after-depolarisations (often denoted ‘EADs’ and ‘DADs’ respectively) [34]. If EADs and DADs occur with sufficient magnitude, they can cause a cell to depolarise and trigger an AP that propagates to neighboring cells. It is not known how many PVCs would need to act in concert to be a focus for ectopic AP generation. Most likely, coordination of multiple cells is required to provide a sufficient signal for a propagating wave of depolarisation. However, even those EADs and DADs that do not reach the threshold for triggering an AP can disrupt the cyclical activation of myocytes by rendering them refractory, and thereby causing them to lose synchrony with their neighbours; a putative mechanism for generating electrical re-entry circuits. In the longer term, spontaneous Ca^2+^ signals can alter gene expression, and lead to deleterious phenotypic remodelling of myocytes [35].

A key finding in the present study was the propensity of PVCs to perpetually display spontaneous Ca^2+^ signals even in the presence of electrical pacing. It would be reasonable to expect that EFS could extinguish spontaneous Ca^2+^ signals by providing regular command pulses, and normalising SR Ca^2+^ content. Consistent with this idea, we observed that EFS could dampen spontaneous Ca^2+^ release, in particular in those cells with relatively low spontaneous Ca^2+^ activity. However, the effectiveness of electrical pacing was determined by the frequency of spontaneous Ca^2+^ signals. In essence, if spontaneous Ca^2+^ release occurred close to the EFS frequency then pacing was either partially successful or unsuccessful. Similar observations have been made using model stimulations of spontaneously active atrial myocytes [36]. The tendency of PVCs to show spontaneous Ca^2+^ signals would certainly contribute to heterogeneous electrical responses within a pulmonary vein [16]. Such heterogeneity could lead to the generation of focal activity that may eventually propagate to the heart and disturb the cardiac cycle. Moreover, events that change the occurrence of spontaneous Ca^2+^ release, such as alteration of Ca^2+^ influx or RyR sensitivity can further decrease the ability of APs to control PVCs and enhance the likelihood of pro-arrhythmic events. Suppressing spontaneous Ca^2+^ signaling in PVCs may therefore provide a plausible target to prevent the inception of AF. Other studies have also suggested clamping of RyR activity as a putative mechanism for controlling pulmonary vein-induced arrhythmogenesis [37]. Our data suggest that VOCC-mediated Ca^2+^ influx is necessary for EFS, but also provides a source of Ca^2+^ for spontaneous Ca^2+^ signaling. In the present study, we did not characterise all putative Ca^2+^ influx mechanisms, in particular those activated by removal/re-addition of extracellular Ca^2+^, but multiple Ca^2+^ influx pathways may be involved, as in smooth muscle [38].

The reason why PVCs have a high propensity to show spontaneous Ca^2+^ signals is unclear. In previous studies of PVCs [39] and atrial myocytes [40] InsP_3_Rs were identified as triggers for spontaneous Ca^2+^ signaling, but these channels did not play a detectable role within the PVCs used in this study. Since SR Ca^2+^ content is known to regulate RyR activation [41], a plausible explanation is that PVCs have a relatively high SR Ca^2+^ content so that their RyRs open spontaneously. In support of this notion, experiments designed to increase SR Ca^2+^ content using KCl, or RyR sensitivity using caffeine, increased spontaneous Ca^2+^ signaling. In addition, it is evident that PVCs have sufficient SR Ca^2+^ to allow spontaneous Ca^2+^ signals to occur for several minutes in a Ca^2+^-free medium. All manoeuvres that provoked PVCs to show more rapid spontaneous events also made the PVCs resistant to electrical pacing. We therefore suggest that if the SR Ca^2+^ content, Ca^2+^ influx or RyR sensitivity are sufficiently enhanced, spontaneous Ca^2+^ activity will be accelerated and the PVCs become insensitive to EFS.

## Supporting Information

Figure S1 **PVCs show spontaneous activity and can be paced by electrical field stimulation (EFS) at 37°C.** The line-scans in **A_i_–_iii_** illustrate the correlation between EFS and contraction in PVCs at 37°C. **(A_i_)** depicts the lack of coordinated contraction in the absence of EFS. Whereas, 1 Hz **(A_ii_)** and 5 Hz **(A_iii_)** EFS caused obvious contraction. Panels **B_i–iii_** illustrate the Ca^2+^ transients observed in absence of EFS **(B_i_)**, and during 1 Hz **(B_ii_)** and 5 Hz **(B_iii_)** EFS. **(C)** The frequency of the spontaneous Ca^2+^ transients is not significantly different at room temperature (RT; *n* = 53 cells, 15 slices) and at 37°C (*n = *57 cells, 14 slices).
(TIF)

Figure S2 **Viability of PVCs within a lung slice.** The images in **A_i_**_–**iii**_ depict a region of a lung slice with PVCs (a portion of them are outlined) and surrounding cells. The lung slice was incubated with Oregon Green BAPTA-1 AM **(A_i_)** and Propidium Iodide (PI) **(A_ii_)** to examine dye retention/exclusion. The images are merged in **(A_iii_)**. The images were taken during Day 1 after the preparation of the slice. The traces in **B** show the spontaneous Ca^2+^ signals in an active PVC (sampled from the region indicated by the blue circle in **A**) and an inactive cell (sampled from the region indicated by the magenta circle in **A**). **C** illustrates that active PVCs maintained a relatively low PI fluorescence over 4 days in culture. In contrast, dead cells (non-identified) within the slices had a relatively higher PI fluorescence. The absolute PI fluorescence sampled from labelled cells did not significantly change over the period of 4 days (One-Way ANOVA, *n = *2–29 cells, 1–10 slices per day).
(TIF)

Figure S3 **Atrial myocytes within atrial slices do not show spontaneous Ca^2+^ transients. (A)** A 2-photon fluorescence image of Oregon Green BAPTA-1-loaded myocytes within an atrial slice. **(B)** Atrial myocytes do not display Ca^2+^ transients without EFS. However, these atrial myocytes showed Ca^2+^ transients in response to 1 Hz **(C)**, 2.5 Hz **(D)** and 4 Hz **(E)** EFS. Ca^2+^ signals were sampled from the region bounded by the dashed box.
(TIF)

Figure S4 **Intracellular Ca^2+^ waves change to intercellular Ca^2+^ waves after removal and re-addition of extracellular Ca^2+^.** **(A)** A line-scan plot illustrating that intracellular Ca^2+^ waves (and subcellular Ca^2+^ signals) remain within individual cells (the cell boundaries are indicated by the dashed lines) under control conditions. **(B)** A line scan plot of the same cellular regions as in A following removal and re-addition of extracellular Ca^2+^ illustrates that the previously constrained Ca^2+^ intracellular waves are transformed into propagating intercellular Ca^2+^ waves (experiment similar to that shown in Fig. 5A).
(TIF)

Figure S5 **Ryanodine inhibits Ca^2+^ signals in PVCs.** **(A)** Ca^2+^ trace and **(B_i_–B_iii_)** line-scan plots showing that 10 µM ryanodine gradually inhibited spontaneous Ca^2+^ signals within PVCs (*n* = 26 cells, 6 slices). **(C)** Ca^2+^ trace and **(D_i_–D_iii_)** line-scan plots of PVCs during 1 Hz EFS. The EFS-evoked responses were initially enhanced, and then progressively inhibited, by 10 µM ryanodine (*n* = 20 cells, 3 slices).
(TIF)

Figure S6 **Effect of a maximal caffeine concentration on PVCs.** **(A)** and **(B)** show representative traces of Ca^2+^ signals caused by superfusion of spontaneously active PVCs with 20 mM caffeine. Caffeine increased the spontaneous Ca^2+^ transient frequency, leading to a maintained plateau of elevated Ca^2+^.
(TIF)

Figure S7 **Nifedipine inhibits EFS-entrained Ca^2+^ signals, thus revealing spontaneous Ca^2+^ signals.** Nifedipine also progressively reduces the frequency of spontaneous Ca^2+^ signals in unpaced cells. **(A)** Ca^2+^ trace and **(B_i_–B_iv_)** line-scan analysis of Ca^2+^ signals in PVCs paced by 1 Hz EFS. The EFS responses are progressively inhibited by 100 µM nifedipine so that spontaneous Ca^2+^ signals become evident. **(C)** Line-scan analysis illustrating that EFS-induced contraction observed under control conditions **(C_i_)** is inhibited by 100 µM nifedipine **(C_ii_)**. **(D)** Ca^2+^ trace and **(E_i_, E_ii_)** line-scan analysis showing a gradual reduction of spontaneous Ca^2+^ signals in response to 100 µM nifedipine. **(F)** Quantitation of the declining frequency of the spontaneous Ca^2+^ signals in nifedipine-treated PVCs (*n* = 21 cells, 5 slices).
(TIF)

Video S1 **Spontaneous and EFS induced contraction of a pulmonary vein.** Phase contrast image of a cross section through a pulmonary vein, showing a small amount of spontaneous contraction around the whole circumference of the vein. After application of EFS a stronger contraction of the pulmonary vein is observed in response to every electric pulse. Image acquisition rate 15 frames per second.
(MP4)

Video S2 **Spontaneous and EFS induced Ca^2+^ signals in PVCs.** The video shows spontaneous Ca^2+^ signaling in Oregon-Green BAPTA-1 loaded PVCs. The spontaneous Ca^2+^ waves either stay intracellular or travel through several PVCs. In response to EFS a simultaneous whole cell Ca^2+^ increase is seen in all PVCs. The spontaneous activity continues in-between the EFS pulses. Image acquisition rate 30 frames per second.
(MP4)

Video S3 **Ca^2+^ signals during a Ca^2+^ removal and re-addition experiment.** The first part of the video shows the spontaneous Ca^2+^ signals in PVCs before Ca^2+^ removal from the superfusion medium. The observed Ca^2+^ waves are predominantly intracellular. The second part of the video shows the absence of spontaneous Ca^2+^ signals after 5 minutes in Ca^2+^ free sHBSS (100 µM EGTA). A gradual increase in Ca^2+^ transients after re-addition of 1.3 mM Ca^2+^ is shown in the third part of the video. In contrast to the spontaneous activity seen in the first part, most of the Ca^2+^ waves after Ca^2+^ re-addition are intercellular Ca^2+^ waves, travelling though several cells. Strong, uncoordinated contractions develop during the Ca^2+^ re-addition period. Image acquisition rate 30 frames per second.
(MP4)
