# Supplementary figures and images for: Spontaneous, Pro-Arrhythmic Calcium Signals Disrupt Electrical Pacing in Mouse Pulmonary Vein Sleeve Cells
Source: PLoS One. 2014 Feb 20;9(2):e88649. doi: 10.1371/journal.pone.0088649 (PMC3930634; doi:10.1371/journal.pone.0088649)

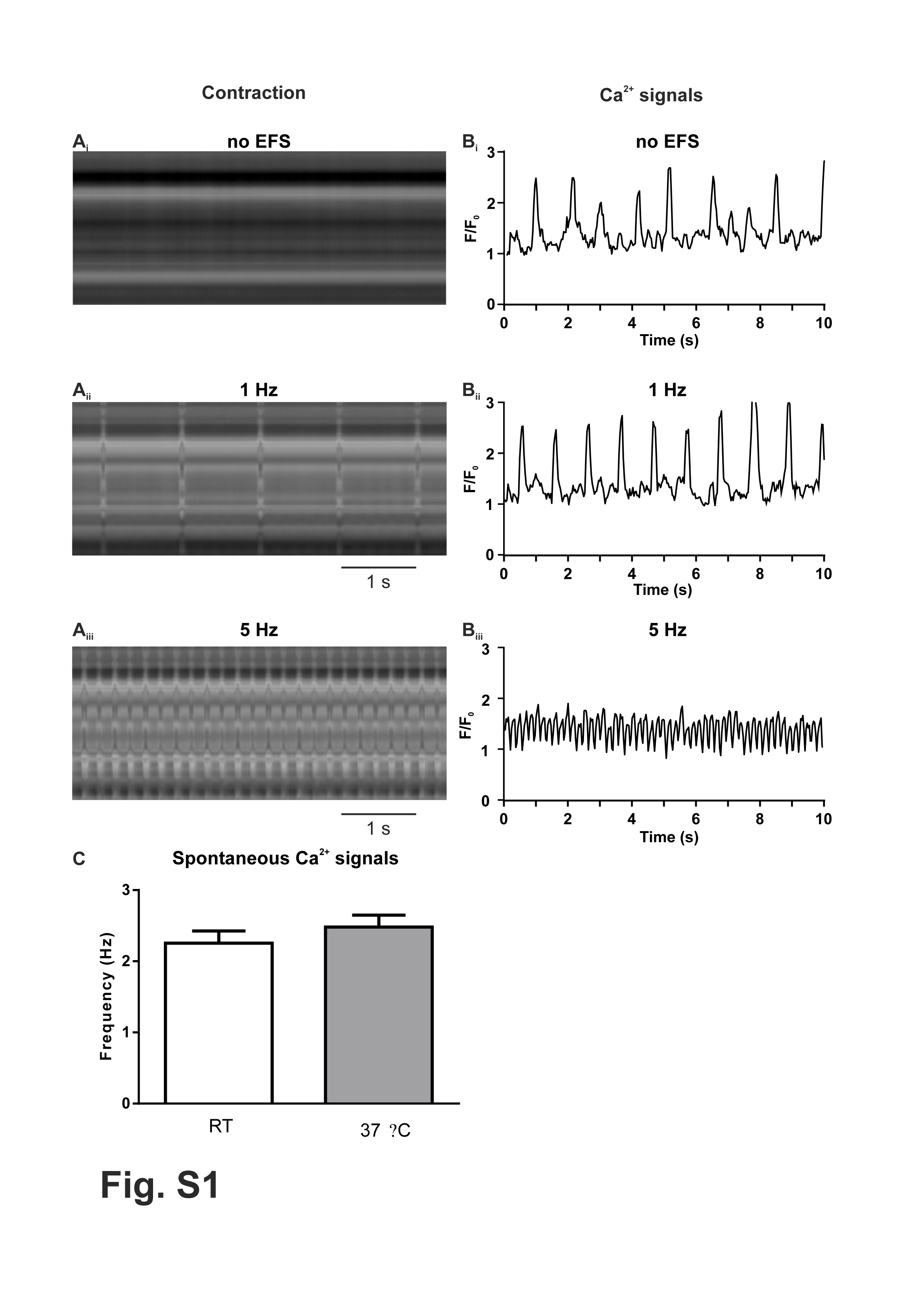

Supplement: Figure S1 — PVCs show spontaneous activity and can be paced by electrical field stimulation (EFS) at 37°C. The line-scans in Ai–iii illustrate the correlation between EFS and contraction in PVCs at 37°C. (Ai) depicts the lack of coordinated contraction in the absence of EFS. Whereas, 1 Hz (Aii) and 5 Hz (Aiii) EFS caused obvious contraction. Panels Bi–iii illustrate the Ca2+ transients observed in absence of EFS (Bi), and during 1 Hz (Bii) and 5 Hz (Biii) EFS. (C) The frequency of the spontaneous Ca2+ transients is not significantly different at room temperature (RT; n = 53 cells, 15 slices) and at 37°C (n = 57 cells, 14 slices). (TIF) [file pone.0088649.s001.tif]

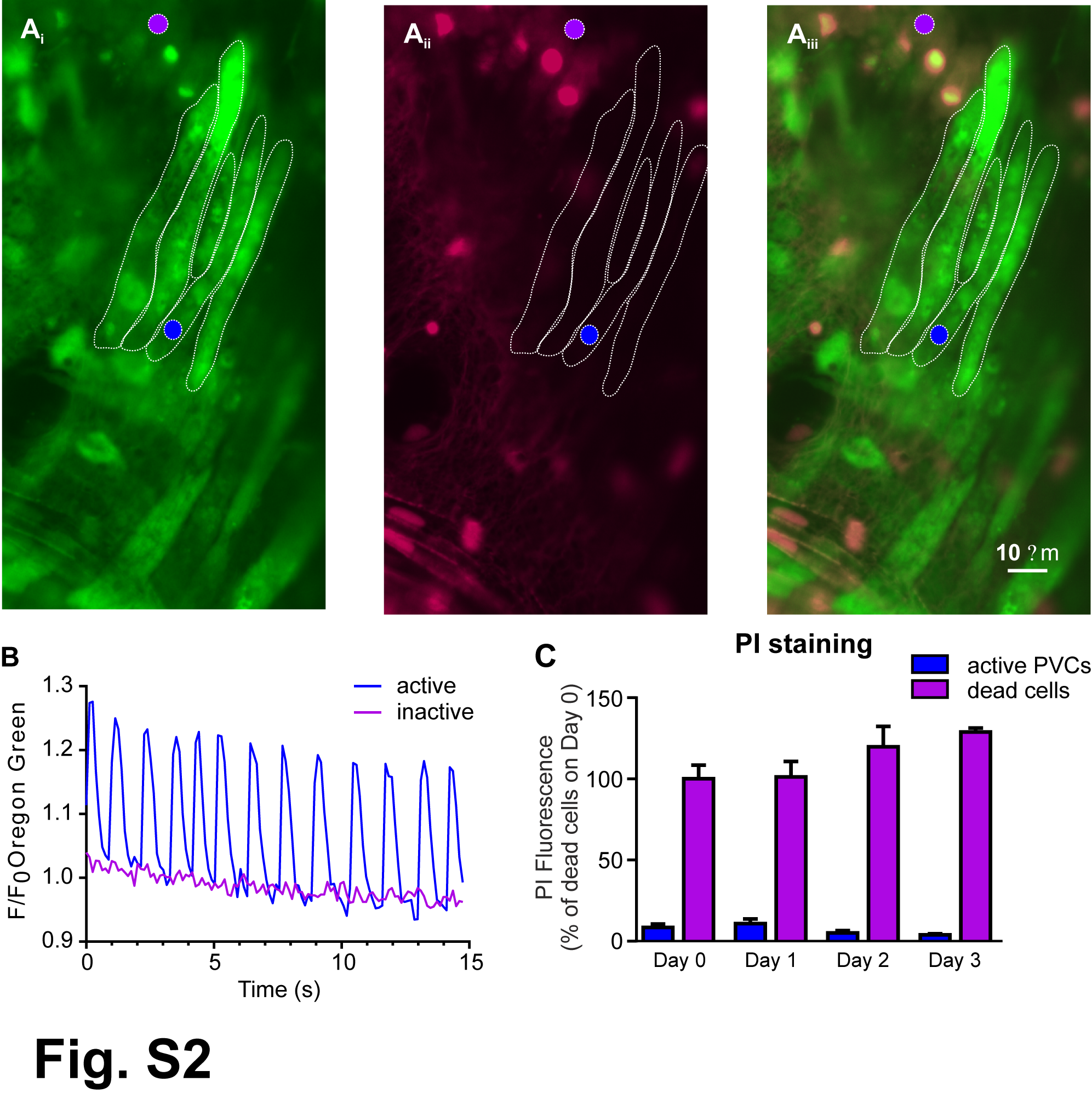

Supplement: Figure S2 — Viability of PVCs within a lung slice. The images in Ai –iii depict a region of a lung slice with PVCs (a portion of them are outlined) and surrounding cells. The lung slice was incubated with Oregon Green BAPTA-1 AM (Ai) and Propidium Iodide (PI) (Aii) to examine dye retention/exclusion. The images are merged in (Aiii). The images were taken during Day 1 after the preparation of the slice. The traces in B show the spontaneous Ca2+ signals in an active PVC (sampled from the region indicated by the blue circle in A) and an inactive cell (sampled from the region indicated by the magenta circle in A). C illustrates that active PVCs maintained a relatively low PI fluorescence over 4 days in culture. In contrast, dead cells (non-identified) within the slices had a relatively higher PI fluorescence. The absolute PI fluorescence sampled from labelled cells did not significantly change over the period of 4 days (One-Way ANOVA, n = 2–29 cells, 1–10 slices per day). (TIF) [file pone.0088649.s002.tif]

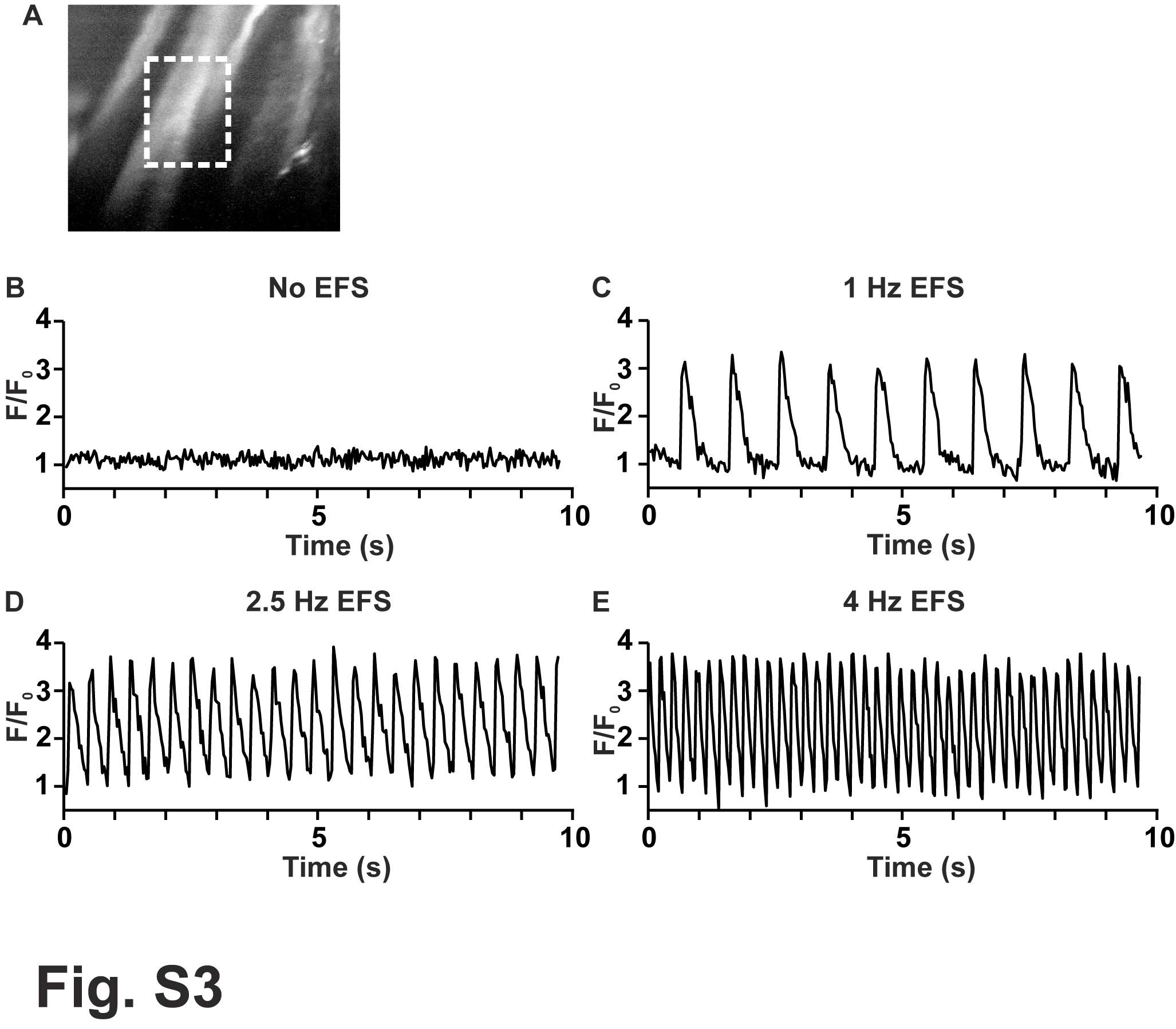

Supplement: Figure S3 — Atrial myocytes within atrial slices do not show spontaneous Ca2+ transients. (A) A 2-photon fluorescence image of Oregon Green BAPTA-1-loaded myocytes within an atrial slice. (B) Atrial myocytes do not display Ca2+ transients without EFS. However, these atrial myocytes showed Ca2+ transients in response to 1 Hz (C), 2.5 Hz (D) and 4 Hz (E) EFS. Ca2+ signals were sampled from the region bounded by the dashed box. (TIF) [file pone.0088649.s003.tif]

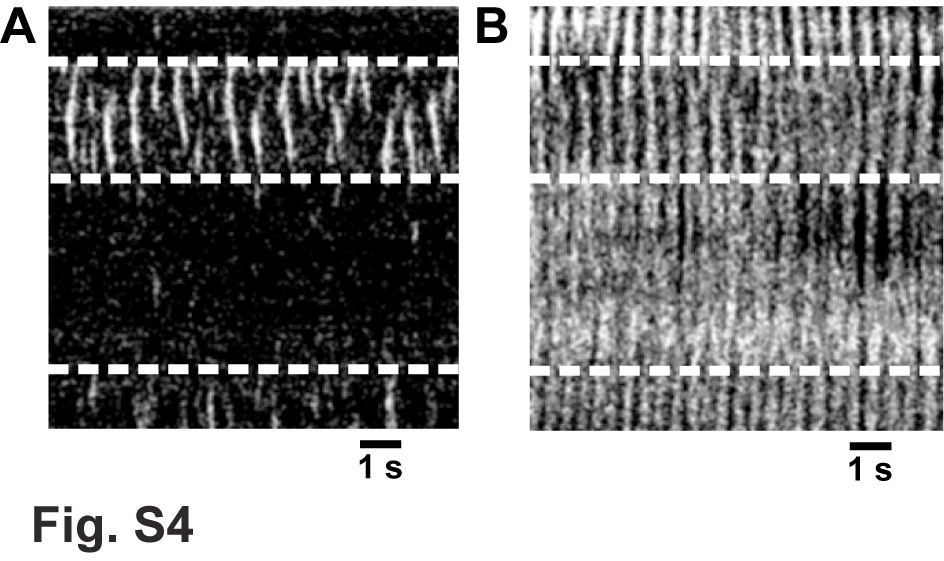

Supplement: Figure S4 — Intracellular Ca2+ waves change to intercellular Ca2+ waves after removal and re-addition of extracellular Ca2+. (A) A line-scan plot illustrating that intracellular Ca2+ waves (and subcellular Ca2+ signals) remain within individual cells (the cell boundaries are indicated by the dashed lines) under control conditions. (B) A line scan plot of the same cellular regions as in A following removal and re-addition of extracellular Ca2+ illustrates that the previously constrained Ca2+ intracellular waves are transformed into propagating intercellular Ca2+ waves (experiment similar to that shown in Fig. 5A). (TIF) [file pone.0088649.s004.tif]

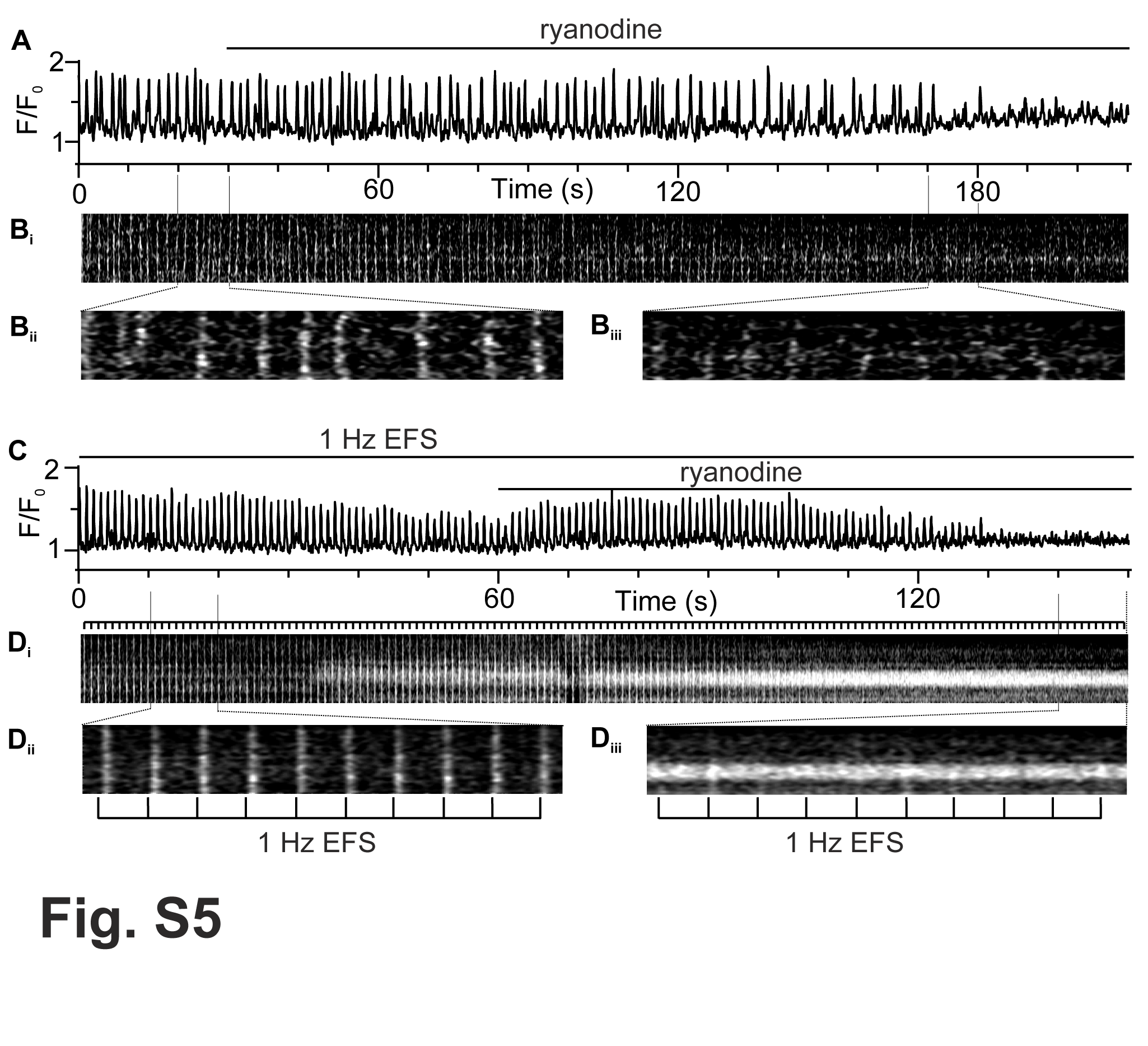

Supplement: Figure S5 — Ryanodine inhibits Ca2+ signals in PVCs. (A) Ca2+ trace and (Bi–Biii) line-scan plots showing that 10 µM ryanodine gradually inhibited spontaneous Ca2+ signals within PVCs (n = 26 cells, 6 slices). (C) Ca2+ trace and (Di–Diii) line-scan plots of PVCs during 1 Hz EFS. The EFS-evoked responses were initially enhanced, and then progressively inhibited, by 10 µM ryanodine (n = 20 cells, 3 slices). (TIF) [file pone.0088649.s005.tif]

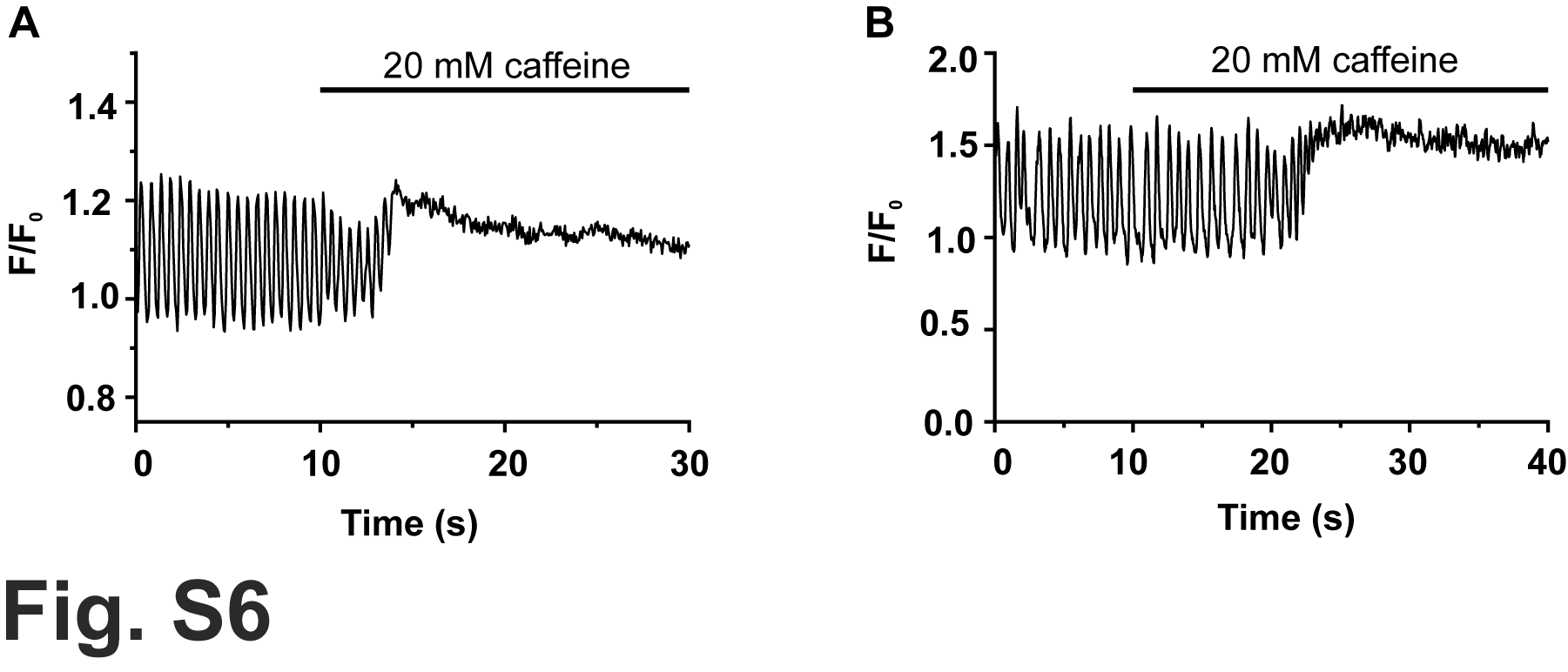

Supplement: Figure S6 — Effect of a maximal caffeine concentration on PVCs. (A) and (B) show representative traces of Ca2+ signals caused by superfusion of spontaneously active PVCs with 20 mM caffeine. Caffeine increased the spontaneous Ca2+ transient frequency, leading to a maintained plateau of elevated Ca2+. (TIF) [file pone.0088649.s006.tif]

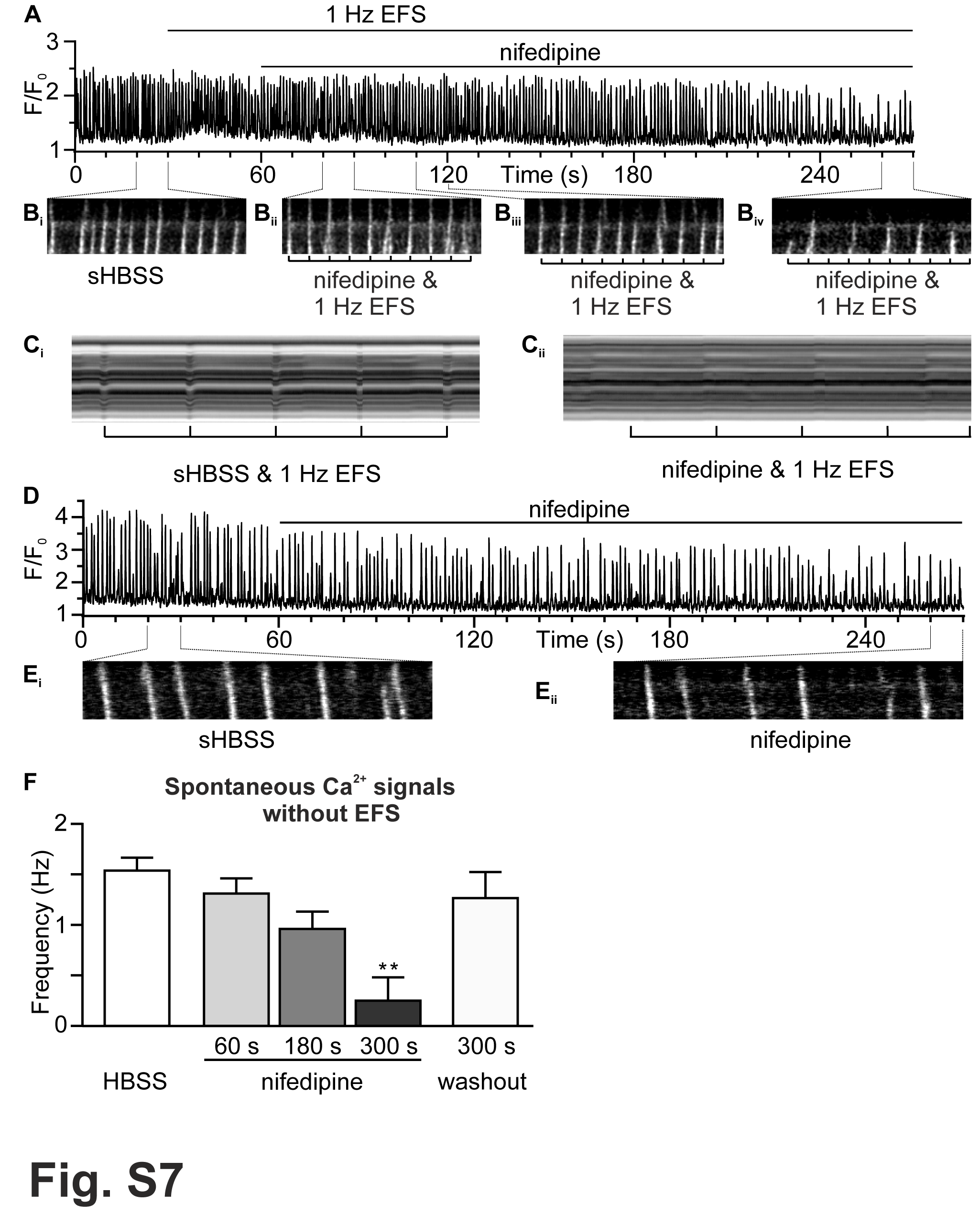

Supplement: Figure S7 — Nifedipine inhibits EFS-entrained Ca2+ signals, thus revealing spontaneous Ca2+ signals. Nifedipine also progressively reduces the frequency of spontaneous Ca2+ signals in unpaced cells. (A) Ca2+ trace and (Bi–Biv) line-scan analysis of Ca2+ signals in PVCs paced by 1 Hz EFS. The EFS responses are progressively inhibited by 100 µM nifedipine so that spontaneous Ca2+ signals become evident. (C) Line-scan analysis illustrating that EFS-induced contraction observed under control conditions (Ci) is inhibited by 100 µM nifedipine (Cii). (D) Ca2+ trace and (Ei, Eii) line-scan analysis showing a gradual reduction of spontaneous Ca2+ signals in response to 100 µM nifedipine. (F) Quantitation of the declining frequency of the spontaneous Ca2+ signals in nifedipine-treated PVCs (n = 21 cells, 5 slices). (TIF) [file pone.0088649.s007.tif]
